# Supplementary material for: ProteinShader: illustrative rendering of macromolecules
Source: BMC Struct Biol. 2009 Mar 30;9:19. doi: 10.1186/1472-6807-9-19 (PMC2672931; doi:10.1186/1472-6807-9-19)
Supplement: Additional file 1 — ProteinShader program without source code. This compressed file contains the complete ProteinShader program including associated libraries, but no source code. A README.txt file gives an overview of the ProteinShader distribution, and the index.html file in the help subdirectory has directions on getting started with the program as well as a set of tutorials. [file 1472-6807-9-19-S1.zip › ProteinShader-beta-0_9_4-binary/help/api/org/proteinshader/structure/enums/package-use.html]

Uses of Package org.proteinshader.structure.enums (ProteinShader API)


|  |  |  |
| --- | --- | --- |
| **Overview** \| **Package** \| Class \| **Use** \| **Tree** \| **Deprecated** \| **Index** \| **Help** | |  |
| PREV   NEXT | **FRAMES**    **NO FRAMES**     **All Classes** |  |


---


## **Uses of Package org.proteinshader.structure.enums**

| Packages that use org.proteinshader.structure.enums | |
| --- | --- |
| **org.proteinshader.graphics** | Holds the drawing classes: Ribbon, Tube, FrenetFrames, Sphere, and Cylinder. |
| **org.proteinshader.graphics.typography** | Holds the classes needed for applying text labels to the curved surfaces of tubes and ribbons. |
| **org.proteinshader.gui.components.controlpanel** | Holds the JPanels used in the right-side retractable control panel of the GUI. |
| **org.proteinshader.structure** | Holds the classes that store information from a Protein Data Bank file: Structure, Model, Chain, AminoAcid, Heterogen, Water, Atom, Bond, Helix, BetaStrand, Loop, *etc*. |
| **org.proteinshader.structure.enums** | Holds the enums needed by org.proteinshader.structure and its subpackages. |
| **org.proteinshader.structure.exceptions** | Holds the exception classes needed by org.proteinshader.structure and its subpackages. |
| **org.proteinshader.structure.io** | Holds the classes needed for reading Protein Data Bank structure files. |
| **org.proteinshader.structure.visitor** | Holds Visitor classes that know how to traverse the hierarchy of objects held by class Structure. |
| **org.proteinshader.structure.visitor.modifiers** | Holds helper classes for the ModifierVisitor class, which is used to modify Drawable objects (Atoms, Bonds, and Segments). |

Classes in org.proteinshader.structure.enums used by org.proteinshader.graphics

****RegionEnum****
  
          Provides a Region type enumeration (Loop, Helix,
or BetaStrand) useful for marking AminoAcids.

Classes in org.proteinshader.structure.enums used by org.proteinshader.graphics.typography

****RegionEnum****
  
          Provides a Region type enumeration (Loop, Helix,
or BetaStrand) useful for marking AminoAcids.

Classes in org.proteinshader.structure.enums used by org.proteinshader.gui.components.controlpanel

****DecorationEnum****
  
          Provides an enumeration to specify if a Segment object should be plain,
with text labels, or with halftoning when the Segment is rendered as a
three-dimensional tube or ribbon.

****ResidueEnum****
  
          Provides an enumeration that can be used to restrict a Visitor to
traversing only a particular Residue type.

****SideChainEnum****
  
          Provides an enumeration to specify how an amino
acid side chain should be displayed is it is shown
in combination with a tube or ribbon segment.

****VisibilityEnum****
  
          Provides an enumeration to specify if a Drawable object is opaque,
translucent, or invisible.

Classes in org.proteinshader.structure.enums used by org.proteinshader.structure

****AminoAcidEnum****
  
          Provides an enumeration of the amino acids found in protein.

****AtomEnum****
  
          Provides an enumeration of atom types: C, H, N, O, S, *etc.*

****BondEnum****
  
          Provides an enumeration for molecular bonds.

****DecorationEnum****
  
          Provides an enumeration to specify if a Segment object should be plain,
with text labels, or with halftoning when the Segment is rendered as a
three-dimensional tube or ribbon.

****DrawableEnum****
  
          Provides an enumeration to specify if a Drawable object is an
Atom, Bond, or Segment.

****HelixEnum****
  
          Provides an enumeration of the helix types specified in the
 Protein Data Bank .

****HelixShapeEnum****
  
          Lists the possible shapes that can be used to represent a Helix.

****RegionEnum****
  
          Provides a Region type enumeration (Loop, Helix,
or BetaStrand) useful for marking AminoAcids.

****SideChainEnum****
  
          Provides an enumeration to specify how an amino
acid side chain should be displayed is it is shown
in combination with a tube or ribbon segment.

****VisibilityEnum****
  
          Provides an enumeration to specify if a Drawable object is opaque,
translucent, or invisible.

Classes in org.proteinshader.structure.enums used by org.proteinshader.structure.enums

****AAColorEnum****
  
          Provides an enumeration of an amino acid color scheme.

****AminoAcidEnum****
  
          Provides an enumeration of the amino acids found in protein.

****AtomEnum****
  
          Provides an enumeration of atom types: C, H, N, O, S, *etc.*

****BondEnum****
  
          Provides an enumeration for molecular bonds.

****CPKColorEnum****
  
          Provides an enumeration of the CPK color scheme that is needed by
AtomEnum.

****DecorationEnum****
  
          Provides an enumeration to specify if a Segment object should be plain,
with text labels, or with halftoning when the Segment is rendered as a
three-dimensional tube or ribbon.

****DrawableEnum****
  
          Provides an enumeration to specify if a Drawable object is an
Atom, Bond, or Segment.

****HelixEnum****
  
          Provides an enumeration of the helix types specified in the
 Protein Data Bank .

****HelixShapeEnum****
  
          Lists the possible shapes that can be used to represent a Helix.

****RegionColorEnum****
  
          PrProvides default colors for regions of protein secondary structure
(alpha-helices, beta-strands and general loop regions).

****RegionEnum****
  
          Provides a Region type enumeration (Loop, Helix,
or BetaStrand) useful for marking AminoAcids.

****ResidueEnum****
  
          Provides an enumeration that can be used to restrict a Visitor to
traversing only a particular Residue type.

****SideChainEnum****
  
          Provides an enumeration to specify how an amino
acid side chain should be displayed is it is shown
in combination with a tube or ribbon segment.

****VisibilityEnum****
  
          Provides an enumeration to specify if a Drawable object is opaque,
translucent, or invisible.

Classes in org.proteinshader.structure.enums used by org.proteinshader.structure.exceptions

****AtomEnum****
  
          Provides an enumeration of atom types: C, H, N, O, S, *etc.*

Classes in org.proteinshader.structure.enums used by org.proteinshader.structure.io

****AtomEnum****
  
          Provides an enumeration of atom types: C, H, N, O, S, *etc.*

****HelixEnum****
  
          Provides an enumeration of the helix types specified in the
 Protein Data Bank .

****RegionEnum****
  
          Provides a Region type enumeration (Loop, Helix,
or BetaStrand) useful for marking AminoAcids.

****VisibilityEnum****
  
          Provides an enumeration to specify if a Drawable object is opaque,
translucent, or invisible.

Classes in org.proteinshader.structure.enums used by org.proteinshader.structure.visitor

****AminoAcidEnum****
  
          Provides an enumeration of the amino acids found in protein.

****AtomEnum****
  
          Provides an enumeration of atom types: C, H, N, O, S, *etc.*

****VisibilityEnum****
  
          Provides an enumeration to specify if a Drawable object is opaque,
translucent, or invisible.

Classes in org.proteinshader.structure.enums used by org.proteinshader.structure.visitor.modifiers

****DecorationEnum****
  
          Provides an enumeration to specify if a Segment object should be plain,
with text labels, or with halftoning when the Segment is rendered as a
three-dimensional tube or ribbon.

****SideChainEnum****
  
          Provides an enumeration to specify how an amino
acid side chain should be displayed is it is shown
in combination with a tube or ribbon segment.

****VisibilityEnum****
  
          Provides an enumeration to specify if a Drawable object is opaque,
translucent, or invisible.

---


|  |  |  |
| --- | --- | --- |
| **Overview** \| **Package** \| Class \| **Use** \| **Tree** \| **Deprecated** \| **Index** \| **Help** | |  |
| PREV   NEXT | **FRAMES**    **NO FRAMES**     **All Classes** |  |


---

# *Copyright © 2007-2008*
